# Microcin J25 Exhibits Inhibitory Activity Against *Salmonella* Newport in Continuous Fermentation Model Mimicking Swine Colonic Conditions

**DOI:** 10.3389/fmicb.2020.00988

**Published:** 2020-05-25

**Authors:** Sabrine Naimi, Séverine Zirah, Menel Ben Taher, Jérémie Theolier, Benoît Fernandez, Sylvie Françoise Rebuffat, Ismail Fliss

**Affiliations:** ^1^STELA Dairy Research Center, Institute of Nutrition and Functional Foods, Université Laval, Québec, QC, Canada; ^2^Laboratoire Molécules de Communication et Adaptation des Microorganismes (MCAM), Muséum National d’Histoire Naturelle, Centre National de la Recherche Scientifique, Paris, France

**Keywords:** microcin J25, reuterin, rifampicin, *Salmonella*, swine colon, PolyFermS model

## Abstract

Microcin J25 (MccJ25), a 21-amino acid bacteriocin produced by *Escherichia coli* (*E. coli*), is a potent inhibitor of Enterobacteriaceae, including pathogenic *E. coli*, *Salmonella*, and *Shigella*. Its lasso structure makes it highly stable and therefore of interest as a possible antimicrobial agent in foods or as an alternative to antibiotics in livestock production. In the present study, we aimed to evaluate *in vitro* the inhibitory activity of MccJ25 against *Salmonella enterica* subsp. *enterica* serovar Newport ATCC 6962 (*Salmonella* Newport) used as a model pathogen under conditions simulating those of the swine proximal colon. The growth inhibition activity of MccJ25 against *Salmonella* Newport was examined in lysogeny broth (LB) and in modified MacFarlane medium that allows miming the swine colonic conditions. The MccJ25 activity was further determined using the Polyfermentor intestinal model (PolyFermS), an *in vitro* continuous fermentation model that permits deciphering the activity of any antimicrobial molecule in real colon fermentation conditions using selected microbiota. It was set up here to simulate the porcine proximal colon fermentation. In these conditions, the inhibition activity of MccJ25 was compared to those of two antimicrobial agents, reuterin and rifampicin. The minimal inhibitory concentration (MIC) of MccJ25 was determined at 0.03 μM in LB medium, compared to 1,079 and 38 μM for reuterin and rifampicin, respectively, showing a significantly higher potency of MccJ25. Total inhibition of *Salmonella* Newport was observed in LB medium over 24 h of incubation at concentrations starting from the MIC. In the PolyFermS model, MccJ25 induced a significantly stronger inhibition of *Salmonella* Newport growth than reuterin or rifampicin. A specific and sensitive LC-MS method allowed to detect and quantify MccJ25 in the PolyFermS fermentation system, showing that MccJ25 remains stable and active against *Salmonella* in conditions mimicking those found in swine colon. This study paves the way for further exploring the potential of this bacteriocin as an alternative to antibiotics in livestock.

## Introduction

Currently generating billions of dollars in export income, pork production is one of the most promising sectors of the world economy. As is the case with other livestock, pigs are sensitive to various infectious diseases, which cause major losses of revenue. Indeed, foodborne gastrointestinal (GI) tract infections in animals have become a major health problem that has reached epidemic proportions, especially in industrialized countries (Scallan et al., 2011; Crim et al., 2015). The increased incidence of these infections is due mainly to bacterial pathogens such as *Salmonella* spp., which are known to be responsible for one of the most widespread enteric diseases in swine. Swine is a major reservoir for *Salmonella enterica*, the principal causative agent of salmonellosis, an acute enteric disease that reduces animal weight gain and increases mortality, thus decreasing productivity (Kim and Isaacson, 2017). *S. enterica* is therefore a major contributor to economic losses in the industry and above all constitutes a menace to human health (Foley et al., 2008). Controlling *Salmonella* has thus become essential in pork production.

Antibiotics have long been used widely as feed additives to enhance livestock growth or to treat bacterial infections including salmonellosis (McEwen and Fedorka-Cray, 2002). In commercial pork production, piglets are weaned early, followed by abrupt introduction to a solid diet. These methods constitute stress factors that most often lead to diarrhea and slow growth. For many years, antibiotics have been often used at this stage to suppress the activity of gut pathogens and thereby ensure growth. However, this practice has led to the emergence of antibiotic resistance in human commensal and pathogenic bacteria, which has raised public concern and intensified the search for alternative feeding strategies as well as alternatives to antibiotics in livestock production. Moreover, the lack of new families of antibiotic molecules put on the market for about 30 years and the total ban on the use of antibiotics as growth promoters in animal feeds in several countries, including those in the European Union (Castanon, 2007), increased the urgency. Given the danger raised by bacterial resistance to antibiotics in humans, and even more in the One Health context now approved by most countries worldwide, this line of research has thus become paramount. In this context, one interesting track for preventing bacterial infections and reducing the incidence of enteric diseases in livestock is the use of natural antimicrobial peptides (AMPs), of which several are produced by bacteria and are called bacteriocins (Ageitos et al., 2017).

Bacteriocins are defined as ribosomally synthesized AMs with molecular masses generally less than 10 kDa (Rea et al., 2011; Hammami et al., 2013). Unlike antibiotics, they are typically active at nanomolar concentrations and can have narrow spectrum of activity, targeting species closely related to the producer species (Nes, 2011). The use of bacteriocins such as nisin (Stahl et al., 2004; Deegan et al., 2006) as natural antimicrobial agents has already been considered in several foodstuffs including dairy products (Benech et al., 2002), meats (Abee et al., 1995; Siragusa et al., 1999), and plant products (Allende et al., 2007). Nisin-based veterinary preparations, namely Wipe-Out^®^, Dairy Wipes and Mast Out^®^ (ImmuCell Corporation) have been available commercially for several years for the prevention of mastitis in dairy cows (Cotter et al., 2005; Pieterse and Todorov, 2010). Added to the chicken feed, nisin improved their daily weight gain and decreased *Bacteroides* and Enterobacteriaceae counts in the ileum of broiler chicken (Józefiak et al., 2013).

However, most of the studies reported so far focus on bacteriocins produced by Gram-positive bacteria, mainly lactic acid bacteria, while only few data have been reported on bacteriocins from Gram-negative bacteria, which are called microcins (Duquesne et al., 2007; Baquero et al., 2019). One of the most studied Gram-negative bacteriocins is microcin J25 (MccJ25). This 21-amino acid peptide produced by *E. coli* inhibits pathogenic Enterobacteriaceae including *Salmonella* since the nanomolar range (Sable et al., 2000; Rintoul et al., 2001; Vincent et al., 2004). This activity results mainly in both *Salmonella* and *E. coli* from binding to the nucleotide triphosphate channel of bacterial RNA polymerase, thus inhibiting transcription (Adelman et al., 2004; Mukhopadhyay et al., 2004; Braffman et al., 2019). A second independent mode of action involves disruption of cytoplasmic membrane energization by targeting the respiratory chain, as shown first for *Salmonella* serovar Newport (Rintoul et al., 2001). It was further demonstrated in *E. coli* that MccJ25 inhibits the activity of respiratory chain enzymes and increases the production of reactive oxygen species (Bellomio et al., 2007; Galván et al., 2019).

The MccJ25 antibacterial activity against *Salmonella* is maintained in biological fluids (Lopez et al., 2007), in different biological food products (Yu et al., 2019) and in a murine model of infection (Lopez et al., 2007). More recently, therapeutic administration of MccJ25 attenuated enterotoxigenic *Escherichia coli* (ETEC) clinical symptoms in a mouse model of intestinal inflammation and showed a reduction of the intestinal pathogen colonization and a decrease of inflammation (Yu et al., 2020). Administration of MccJ25 to poultry or weaned pigs, either via a probiotic strain engineered to produce MccJ25 (Forkus et al., 2017), or via diet supplementation by MccJ25 (Yu et al., 2017; Wang et al., 2020), was shown to effectively reduce *S. enteritidis* carriage (Forkus et al., 2017), or improve growth performance and gut health (Yu et al., 2017; Wang et al., 2020). However, very little information is available on the stability and activity of MccJ25 under the conditions encountered specifically in the human or animal gastrointestinal tract. Previously, our group described the degradome of MccJ25 in conditions mimicking the upper portion of the human gastrointestinal tract (i.e., stomach, duodenum, and ileon), which can be obtained using a dynamic simulator model (TIM-1). This study showed high stability of MccJ25 in the stomach and partial degradation by the enzymes encountered in the duodenum (Naimi et al., 2018). However, nothing is known about the stability and activity of MccJ25 in the colon and particularly in the proximal colon, a major site of salmonellosis in pigs.

Indeed *in vivo* studies are particularly efficient to provide information on the activity and toxicity of bioactive compounds such as antimicrobials in animal models and gut. However, they have to face both high costs and ethical concerns when *in vivo* infection trials have to be conducted or several conditions should be compared. Moreover, they lead to complex effects involving host responses that are in some cases difficult to unravel. Therefore, *in vitro* gut fermentation models can circumvent these inconveniences, while miming specific colon conditions. As batch fermentations proved their limitation in terms of dependence on the inoculate density and experiment duration, a continuous fermentation model involving an immobilization process for entrapment of fecal microbiota, which thus prevents the instability of the microbiota community diversity, has been developed. The so called PolyFermS (Zihler Berner et al., 2013; Tanner et al., 2014a) has been proved to have high stability and reproducibility. It was successfully applied previously to different intestinal models, including colon microbiota of children, elderly, or pigs (Zihler Berner et al., 2013; Tanner et al., 2014a; Fehlbaum et al., 2015, 2016) and was selected here.

The present study aims at providing data on MccJ25 stability and activity against *Salmonella* under colonic conditions. Using the PolyFermS *in vitro* continuous fermentation model to mimick the conditions of the swine proximal colon, we show here that MccJ25 maintains its potent antibacterial activity against *Salmonella enterica* subsp. *enterica* serovar Newport, one of the pathogens most frequently identified in swine. The activity exhibited by MccJ25 in these conditions is compared to those exerted in similar conditions by two broad spectrum antimicrobial compounds, reuterin produced by several *Lactobacillus reuteri* strains used as probiotics (Cleusix et al., 2007; Schaefer et al., 2010) and the conventional antibiotic rifampicin.

## Materials and Methods

### Bacterial Strains

*E. coli* MC4100 harboring the plasmid pTUC202 obtained from the MCAM laboratory collection (Muséum national d’Histoire naturelle, Paris, France) was used for the production of MccJ25. *Lactobacillus reuteri* ATCC 53608 purchased from American Type Culture Collection (ATCC, Manassas, VA, United States) was used for the production of reuterin. *Salmonella enterica* subsp. *enterica* serovar Newport ATCC 6962 (hereinafter called *Salmonella* Newport), the test strain for antibacterial activity assays, was purchased from Microbiologics Inc. (St. Cloud, Minnesota, United States). Both *E. coli* MC4100 pTUC202 and *Salmonella* Newport were cultured overnight at 37°C in LB (also called Luria-Bertani medium, LB) (tryptone 10 g/L, yeast extract 5 g/L, sodium chloride 10 g/L; Difco, Sparks, MD, United States; batch 244620), under aerobic condition.

### Production of MccJ25 and Reuterin

MccJ25 was produced from 1.5 L cultures of *E. coli* MC4100 pTUC202, as described previously (Ducasse et al., 2012). Briefly, the culture supernatant was submitted to solid-phase extraction on a Sep-Pak C18 35 cc cartridge (Waters, Milford, United States). MccJ25 was eluted with acetonitrile/water (30% v/v) containing 0.1% HCl and further purified by reversed phase high-performance liquid chromatography (RP-HPLC) (Beckman Coulter System Gold, Mississauga, ON, Canada) on a preparative C18 column (Luna 10 μm, 100 Å, 21.10 × 250 mm, Phenomenex, CA, United States) at a flow rate of 10 mL/min using a 25–100% linear gradient of filtered acetonitrile/0.18% HCl in ultra-pure water (PureLab Ultra, ELGA, United States) with detection at 214 nm. Purified MccJ25 was lyophilized and stored at −20°C until use.

Reuterin (3-hydroxypropionaldehyde, 3-HPA) was produced from *Lactobacillus reuteri* ATCC 53608 and purified, according to a protocol adapted from previously published ones (Doleyres et al., 2005; Cleusix et al., 2007). Briefly, MRS broth medium (Nutri-Bact; catalog number QB-39-2285) supplemented with 20 mM glycerol was inoculated (1% v/v) and incubated anaerobically at 37°C for 16 h. The culture was centrifuged (1500 g, 10 min, 20°C) and the cells were washed with 0.1 M potassium phosphate buffer (pH 7.0), centrifuged again, re-suspended in 300 mM glycerol solution and incubated anaerobically at room temperature for 45 min. This suspension was centrifuged (15,000 *g*, 5 min, 4°C), filtered (0.22 μm, Millipore) and lyophilized. Reuterin was then purified according to (Cleusix et al., 2007) on a silica gel 60 (0.060–0.2 mm, 70–230 mesh; Alfa Aesar) preparative chromatography column (2.8 × 35 cm, Bio-Rad Econo-Column), with acetone:ethyl acetate (2:1) as eluent at a flow rate of 5 mL/min. The concentration of reuterin was determined by colorimetric method using acrolein (Lüthi-Peng et al., 2002). A linear standard curve with known initial concentrations of acrolein (0.001–1.0 M) was generated. Then, 750 μL of 10 mM tryptophan dissolved in 1.8% HCl were added to 3 mL of 37% HCl. For reuterin quantification, a 1 mL sample was mixed with 0.75 mL of tryptophan solution and 3 mL of HCl 37%. Mixtures containing samples and standards were incubated for 20 min at 37°C and the absorbance at 560 nm was measured on an Infinite R F200 Pro microplate reader (Tecan Inc., NC, United States). Reuterin samples were diluted with distilled water before mixing with reagents to ensure a final OD_560_ < 1 (Doleyres et al., 2005).

Rifampicin (purity ≥ 97%, HPLC) was purchased from Sigma-Aldrich (St. Louis, MO, United States) and stored at −20°C until use.

For *in vitro* inhibition assays, lyophilized MccJ25 was solubilized in sterile distilled water supplemented with 5% acetonitrile, which proved its lack of toxicity for tested bacteria. Reuterin was directly used from the prepared solution and rifampicin was dissolved in sterile distilled water.

### Modified Macfarlane Medium

A nutrient medium described previously and used to simulate conditions of the human colon (Macfarlane et al., 1998) was modified as described previously (Tanner et al., 2014a) to obtain carbohydrate and protein concentrations close to the swine ileal chyme composition, and thus simulating the conditions in the swine proximal colon (Supplementary Table S1). The medium was set at pH 6.0, autoclaved at 121°C for 20 min and stored at 4°C until use. Vitamins and L-cysteine HCl were added separately in filter-sterilized (0.22 μm, Millipore) solutions (Michel et al., 1998) for *in vitro* antimicrobial activity assays.

### Antibacterial Activity Measurement and Determination of Minimal Inhibitory Concentrations (MIC) and Minimal Bactericidal Concentrations (MBC)

A micro-dilution method described in the Clinical and Laboratory Standards Institute (CLSI) guidelines was used for the determination of MIC and MBC of the different antimicrobial compounds. Two fold serial dilutions of MccJ25 (0.475 mM), reuterin (138.1 mM), or rifampicin (1.215 mM) were prepared in sterile flat-bottom 96-well polystyrene micro-plates (BD Labware, Franklin, NJ, United States) starting with 125 μL of antimicrobial solution in 125 μL of LB medium. These were inoculated with 50 μL of an overnight culture of *Salmonella* Newport diluted to 5 × 10^5^ CFU/mL. After incubation for 24 h at 37°C, the optical density at 595 nm was recorded using an Infinite R F200 Pro microplate reader (Tecan Inc., NC, United States). MICs and MBCs were defined as the lowest concentration that inhibited *Salmonella* Newport growth completely, based on optical density measurement, and the lowest concentration at which no colony appeared on agar after 72 h of incubation, respectively.

### Bioavailability of the Residual MccJ25

In order to determine whether or not MccJ25 remains stable and active against *Salmonella* Newport after incubation in LB broth or in modified Macfarlane broth, the residual activity was tested using the agar diffusion assay. Inhibition of *Salmonella* Newport by MccJ25 was further evaluated using LB broth and modified Macfarlane broth in 24-well micro-plates. Two fold serial dilutions of MccJ25 solution (0.949 mM) in the tested medium (1.5 mL each) were inoculated with 600 μL of an overnight culture of *Salmonella* Newport diluted in LB broth to 5 × 10^5^ CFU/mL. Plates were incubated aerobically at 37°C for 18 h. 500 μL in 24-well micro-plates, either in LB broth or modified Macfarlane broth, were then centrifuged (5,000 g, 10 min, 4°C). The filtered supernatant (0.22 μm, Millipore) was used to evaluate the inhibitory activity of the residual MccJ25 by the agar well diffusion method (Tagg et al., 1976). Hundrerd fifty micron liter of an overnight culture of *Salmonella* Newport were suspended in 25 mL of LB 0.8% agar. Previously purified MccJ25 (0.475 mM) and LB broth were used as positive and negative controls, respectively. Inhibition zone diameters were measured after 18 h of incubation at 37°C.

### PolyFermS Fermentation Model of the Swine Colon

#### Immobilization of Fecal Bacteria

A fecal sample from a healthy adult pig raised under farm conditions and not given any antibiotic during the previous 3 months was collected in a sterile 50 mL Falcon tube. Anaerobiosis was maintained in a Gas-Pak anaerobic jar (Oxoid, Thermo Fisher Scientific) during transport to the laboratory and until the immobilization procedure (Tanner et al., 2014a). About 20 g were suspended in 80 mL of 0.1% peptone water (Difco Laboratories) containing 0.05% L-cysteine-HCl (Sigma-Aldrich, St. Louis, MO, United States) at 37°C and homogenized for 5 min at 200 rpm using a Stomacher (Seward model 400, Norfolk, United Kingdom). The liquid portion was recovered using a serological pipette and centrifuged for 1 min at 700 g to remove large particles. The immobilization procedure begun in an anaerobic chamber (model 1025, Forma Scientific, Marietta, OH, United States) by mixing 20 mL of supernatant into 1 L of a solution of 2.5% w/v gellan, 0.25% w/v xanthan and 0.2% w/v sodium citrate (Cinquin et al., 2004). Beads 1–2 mm in diameter were then formed by pouring this mixture into rapidly stirred canola oil in a flask placed on ice and allowing them to settle for 5 min before removing the canola oil. A 0.1 M CaCl_2_ solution was then added to harden the beads. After stirring for 30 min, the beads were washed with a 0.27 M KCl/0.03 M CaCl_2_ solution and sieved. Gel Beads were recovered and about 75 g were transferred to the inoculum reactor (BIOSTAT^®^ Q plus, Sartorius AG, 79 Göttingen, Germany) containing 250 mL of fresh fermentation medium.

#### Fermentation Procedure

The fermentation-based *in vitro* model of the swine colon has been described elsewhere in detail (Tanner et al., 2014a). The PolyFermS model consisted of a two-stage system comprising a total of three reactors (BIOSTAT^®^ Q plus, Sartorius AG, 79 Göttingen, Germany) operated under conditions of the swine proximal colon (38°C, pH 6.0 with anaerobic conditions maintained by continuous flow of pure CO_2_ through the medium). The modified Macfarlane medium (Supplementary Table S1), which simulates the conditions in the swine proximal colon (Tanner et al., 2014a) was fed to the reactors. Seeded with 30% (v/v) swine fecal beads, the inoculum reactor IR (250 mL) was fed fresh nutrient medium continuously and fed test reactors TR1 and TR2, which also received fresh medium such that their continuous feed was 10% inoculum broth and 90% fresh medium. This required half of the IR effluent; the other half of which was discarded. The mean retention time in the test reactors was 9 h. The feed rate of the three reactors was controlled using peristaltic pumps (model 120U, Watson-Marlow, Falmouth, Cornwall, United Kingdom). The continuous culture was carried out for 35 days. For the first 3 days, the IR was run in fed-batch mode, the medium replaced every 12 h with fresh medium, in order to stabilize fecal bead colonization. Continuous feeding was then started, followed by 12 days of stabilization before connection to TR1 and TR2. Continuous culture was split into three periods: stabilization, treatment and wash. Conditions were kept constant in IR to assess the temporal stability of the system without any manipulation during the entire fermentation, while TR1 and TR2 were subjected to treatments for various periods. Between each period, culture was flushed from TR1 and TR2 as described previously (Tanner et al., 2014a). After each flush, they were stabilized for 3 days until a steady state was reached before starting the next treatment period.

Performed simultaneously in TR1 and TR2, the four treatments consisted in adding *Salmonella* Newport alone at an initial concentration of 10^7^ CFU/mL to each reactor, followed by adding *Salmonella* Newport at this concentration along with each tested antimicrobial compound: 0.397 mM MccJ25, 4 mM reuterin or 0.608 mM rifampicin. For each treatment, samples of test reactor effluent were collected at 0, 2, 4, 6, 8, 10, 12, 24, 48, and 72 h for analysis and stored at −80°C.

### PMA-qPCR

#### Treatment With Propidium Monoazide

The inhibitory activity of MccJ25, reuterin and rifampicin against *Salmonella* Newport was quantified using propidium-monoazide-coupled quantitative polymerase chain reaction (PMA-qPCR). Samples collected from the reactors were treated with propidium monoazide (PMA dye, Biotium, Inc., Hayward, CA, United States) for viable bacteria counts as described previously by Fernandez et al. (2016). Briefly, 2.5 μL of 20 mM PMA solution (1 mg of PMA dissolved in 91 μL of 20% dimethylsulfoxide, stored at −20°C) were added to each 1 mL sample collected from the reactors to obtain a final concentration of 50 μM. Samples were kept for 5 min at room temperature in the dark with occasional vortex mixing, then placed for 5 min on ice 20 cm below a 500 W halogen lamp. Samples were then frozen and stored at −80°C until DNA extraction.

#### DNA Extraction

DNA was extracted from samples of fermentation broth using the PowerSoil^TM^ DNA Isolation Kit (MO BIO Laboratories, CA, United States). Samples were thawed and centrifuged for 10 min at 12,000 g and the pellets were washed twice in Tris-EDTA buffer (10 mM Tris-HCl, 1 mM EDTA). Subsequent steps were performed following the PowerSoil kit instructions.

#### Real-Time PCR Analysis

The inhibitory activity of MccJ25, reuterin and rifampicin against *Salmonella* Newport in the PolyFermS model was evaluated using the quantitative polymerase chain reactions (PCR) for the amplification of 16S rDNA that was performed in MicroAmp^®^ Fast Optical 96-Well Reaction Plates with Barcode (Life Technologies Inc., Burlington, ON, Canada) on an ABI 7500 Real-Time PCR System (Applied Biosystems, Streetsville, ON, Canada). Each sample was analyzed in duplicate. *Salmonella* Newport growth was quantified using the amplification primers invAF (5′-CGTTTCCTGCGGTACTGTTAATT-3′) and invAR (5′-TCGCCAATAACGAATTGCCCGAAC-3′) (Li and Chen, 2013). Extracted DNA was diluted 1/10 (v/v) in DNase-free water (Invitrogen) and placed in the microplate wells (5 μL of diluted extract per well). Each well also contained 12.5 μL of Fast SYBR^®^ Green Master Mix (Applied Biosystems, Burlington, ON, Canada), 1 μL of each primer, forward and reverse, at a final concentration of 5 μM (Sigma-Aldrich, St. Louis, MO, United States) and 5.5 μL of DNase-free water.

### Agar Diffusion Assay for the Evaluation of Antibacterial Activity

Fermentation broth (500 μL samples) collected from bioreactors after treatment with MccJ25, reuterin or rifampicin at 0, 4, 8, 12, and 24 h was centrifuged at 10,000 g for 10 min. The supernatant was filtered through a 0.2 μm syringe filter (VWR, Mississauga, ON, Canada) then tested for inhibitory activity in LB medium (0.75% agar w/v) seeded with 150 μL of an overnight culture of *Salmonella* Newport using the agar well diffusion method as described previously (Wolf and Gibbons, 1996). Wells 7 mm in diameter were cut in the solidified agar (25 mL in standard Petri plates) and filled with 80 μL of test sample. Plates were incubated aerobically at 37°C for 18 h, and the diameters of the inhibition zones were measured.

### Stability of MccJ25 Under Swine Colonic Conditions in the PolyFermS Model

#### Sample Preparation for LC-MS Analysis

Fermentation broth samples were centrifuged at 6,000 g for 10 min at 4°C. Supernatants were micro-filtered (0.22 μm) and kept frozen at −80°C until analysis. Defrosted supernatants were extracted using Oasis^®^ HLB SPE cartridges (30 mg, 1 cc). The cartridge was conditioned with 3 mL of methanol, 3 mL of acetonitrile and 3 mL of 0.1% formic acid solution in Milli-Q water, loaded with 500 μL of supernatant, washed with 3 mL of 0.1% formic acid solution and then eluted with 1 mL 0.1% formic acid solution/acetonitrile 20:80 (v/v). The eluted fractions were vacuum-dried, re-suspended in 0.1% formic acid solution/acetonitrile 90:10 (v/v).

#### LC-MS Analysis

The fermentation broth extracts (5 μL) were analyzed on an ultra-high-performance liquid chromatography system (Ultimate 3000 RSLC, Thermo Scientific) connected to a high-resolution electrospray ionization – quadrupole – time of flight (ESI-Q-TOF) mass spectrometer (Maxis II ETD, Bruker Daltonics). Separation was achieved on an Acclaim RSLC Polar Advantage II column (2.2 μm, 2.1 × 100 mm, Thermo Scientific) at a flow rate of 300 μL/min, using the following gradient of solvent A (milliQ water/0.1% formic acid) and solvent B (HPLC-MS grade acetonitrile/0.08% formic acid) over a total run time of 17.5 min: 5 min at 0%B followed by a linear increase from 0% B to 60% B for 12 min, linear increase to 100% B for 0.2 min, decrease to 0% B for 0.5 min. The MS spectra were acquired in positive ion mode in the mass range m/z 60–1300. The source parameters were as follows: nebulizer gas 35 psi, dry gas 8 L/min, capillary voltage 3500 V, end plate offset 500 V, temperature 200°C. The ESI-Q-TOF instrument was externally calibrated before each run using a sodium formate solution consisting of 10 mM sodium hydroxide in isopropanol/0.2% formic acid (1:1, v/v). A quality control (QC) consisting of a mix of all samples and a blank (injection of water) was recorded every 10 samples to monitor separation quality and absence of cross-contaminations. The data were processed with Data Analysis 4.0 (Bruker Daltonics). MccJ25 quantification was performed from using standard curve of MccJ25 spiked in sterile distilled water at concentrations from 0.062 to 1 mg/mL.

## Results

### Production of MccJ25 and Reuterin

MccJ25 was produced by cultivation of *E. coli* MC4100 pTUC202 in minimal medium M63 and purified from the culture broth by solid phase extraction and RP-HPLC (Supplementary Figure S1A). Recovery of about 45 mg of pure MccJ25 from 1.5 L culture, corresponding to a yield of 58%, was obtained (Supplementary Table S2). An increase of about 3-fold in the specific activity of MccJ25 against *Salmonella* Newport was obtained during the purification procedure.

Two antimicrobials were selected to compare the activity exerted by MccJ25 in the PolyFermS conditions. Rifampicin was selected although tylosin and chlortetracycline are the two antibiotics the most commonly used as growth promoters and for therapeutic purposes in pork production (Apley et al., 2012), as similar to MccJ25 it exerts its antibiotic activity through a mechanism involving interaction with RNA polymerase and inhibition of transcription (Campbell et al., 2001; Adelman et al., 2004; Mukhopadhyay et al., 2004; Alifano et al., 2015). Reuterin, which is naturally produced by *Lactobacillus reuteri* from degradation of glycerol (Cleusix et al., 2007), was also selected. Indeed, it constitutes a good reference for *in vitro* studies both in conventional assays and gut simulating models, due to its limited impact on the gut microbiota despite a broad spectrum antimicrobial activity (Shornikova et al., 1997; Casas and Dobrogosz, 2000).

Reuterin was produced by *Lactobacillus reuteri* ATCC 53608 according to the two-step procedure developed previously (Doleyres et al., 2005). The concentration of reuterin from 300 mM of glycerol was about 138 mM. Pure reuterin free of the accompanying contaminants, essentially glycerol and 1,3 propanediol, was recovered with a yield of 46% using HPLC (Supplementary Figure S1B).

### Inhibition of *Salmonella* Newport by MccJ25, Reuterin and Rifampicin in LB Broth

**FIGURE 1:**
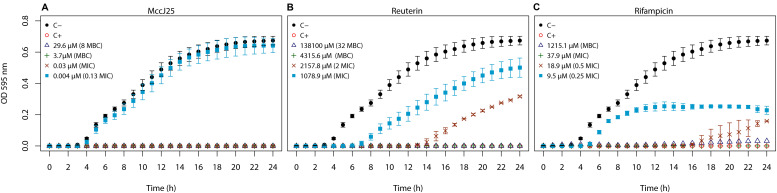
Growth of *Salmonella enterica* subsp. *enterica* serovar Newport ATCC 6962 in 96 well micro-plates in LB broth supplemented with **(A)** MccJ25 at 29.6 μM (8 × MBC) **△**, 3.7μM (MBC) **+**, 0.03 μM (MIC) **×** and 0.004 μM (0. 13 MIC) 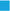, **(B)** reuterin at 138100 μM (32 MBC) **△**, 4315.6 μM (MBC) **+**, 2157.8 μM (2 MIC) **×** and 1078.9 μM (MIC) 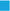, or **(C)** rifampicin at 1215.1 μM (MBC) **△**, 37.9 μM (MIC) **+**, 18.9 μM (0.5 MIC) **×** and 9.5 μM (0.25 MIC) 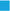. Optical density was measured at 595 nm. C-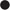 is the negative control (LB broth only) and C+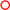 the positive control (LB broth **+** MccJ25 at 474.6 μM). Data are means of two independent experimental repetitions.

LB medium was used to obtain the reference values of minimal inhibitory and minimal bactericidal concentrations (MIC and MBC) of the three antimicrobials against *Salmonella* Newport for the present study. The *Salmonella* Newport growth curves were obtained following incubation in LB in 96-well plates (Figure 1), MIC, and MBC values were determined (Table 1). *Salmonella* Newport appeared highly sensitive to MccJ25 (MIC and MBC at 0.03 and 3.71 μM, respectively), which was a much more potent efficacy than those of reuterin and rifampicin. The MBC/MIC ratio of MccJ25 was 124, which is in favor of a bacteriostatic mechanism of action, according to the interpretation suggested in the CLSI guidelines (Peterson and Shanholtzer, 1992; Pankey and Sabath, 2004). MIC and MBC values were further compared to those determined in unfermented and fermented Macfarlane media further used in the study. MccJ25 remained much more active against *Salmonella* Newport than reuterin and rifampicin in both media, although higher concentrations than in LB were needed. Complete inhibition was observed at 474 M (128 times the MBC) over 4 h and at 237 M (64 times the MBC) over 14 h in unfermented and fermented Macfarlane media, respectively (data not shown). Indeed, higher activity was noticed in the fermented medium where nutrients are scarce and non-specific interactions between MccJ25 and different components of the complex Macfarlane medium are reduced.

**TABLE 1:** Minimal inhibitory concentrations (MIC) and minimal bactericidal concentrations (MBC) of MccJ25, reuterin and rifampicin in LB broth using *Salmonella enterica* subsp. *enterica* serovar Newport (ATCC 6962) in 96-well micro-assay plates.

**MccJ25**			**Reuterin**			**Rifampicin**		
**MIC (μM)**	**MBC (μM)**	**MBC/MIC ratio**	** MIC (μM)**	**MBC (μM)**	**MBC/MIC ratio**	**MIC (μM)**	**MBC (μM)**	**MBC/MIC ratio**
0.03	3.71	124	1078.91	4315.63	4	37.97	1215.15	32

**MIC is the lowest concentration that inhibited S. Newport growth completely, based on optical density measurement. MBC is the lowest concentration at which no colony appeared on agar after 72 h of incubation.**

### Stability of MccJ25 During Inhibitory Activity Assays in LB and Modified Macfarlane Broth

The Macfarlane fermentation medium has been initially optimized and described as simulating the human colon conditions (Macfarlane et al., 1998). To better simulate the conditions in the swine proximal colon, a nutrient medium using corn starch and soy peptone with modified carbohydrate and protein concentrations was further described by Tanner et al. (2014a). This last medium (Supplementary Table S2) was used in the present study and termed “modified Macfarlane medium.”

In order to determine whether or not MccJ25 remains stable and active against *Salmonella* Newport after incubation in modified Macfarlane broth, the residual activity was tested using the agar diffusion assay and compared to that exhibited in LB broth. At a concentration corresponding to the MBC, MccJ25 remained active even after 18 h of incubation in both LB and modified Macfarlane media (Supplementary Figure S2).

### Inhibition of *Salmonella* Newport During Colonic Fermentation in the PolyFermS Model

**FIGURE 2:**
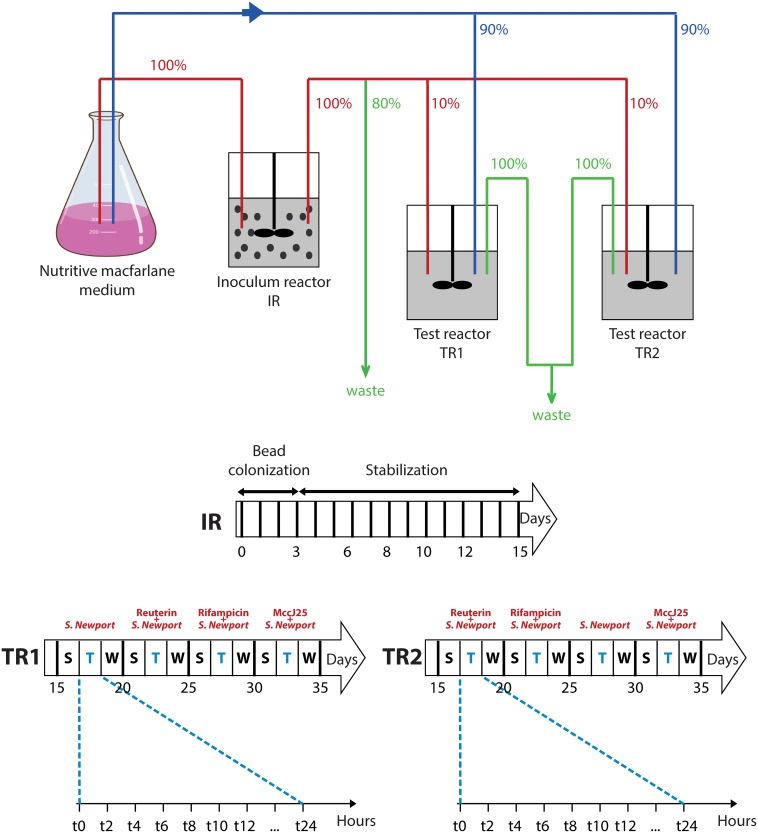
PolyFermS model set-up and experimental treatment schedule. Relative flow rates are indicated in percentage. IR, inoculum reactor containing pig fecal bacteria immobilized in gel beads (30% v/v); TR1, TR2, test bioreactors; S, stabilization period; T, treatment period; W, wash period.

**FIGURE 3:**
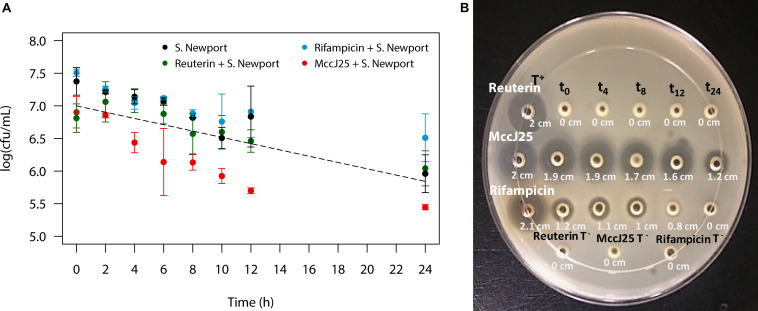
Quantification of the inhibitory activity of MccJ25 against *Salmonella enterica* subsp. *enterica* serovar Newport ATCC 6962 under swine colonic fermentation in the PolyFermS model. **(A)** Washout of *Salmonella* Newport from continuous culture of porcine colonic microbiota in the PolyFermS model starting from 10^7^ CFU/mL. *Salmonella* Newport washout over 24 h as quantified by PMA-qPCR, in absence of antimicrobial compound (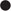) and in presence of MccJ25 at a concentration of 0.397 mM (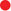), reuterin at 4 mM (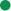) and rifampicin at 0.608 mM (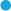). The dashed line represents the theoretical washout of inert particles starting at 10^7^ per mL. Error bars indicate standard deviation based on two independent repetitions in test reactors 1 and 2. Plotted values are based on PMA-qPCR analysis. **(B)** Agar diffusion assay showing the inhibitory activity of MccJ25, reuterin and rifampicin against *Salmonella* Newport over 24 h of culture in modified Macfarlane broth in test reactor 1 (TR1) of the PolyFermS system. T + is the positive control (0.475 mM MccJ25).

The PolyFermS system was seeded with swine microbiota originated from a fresh fecal sample got from a healthy adult animal deprived of antibiotics and raised under farm conditions. The continuous fermentation was carried out for 35 days using the experimental set-up shown in Figure 2. *Salmonella* Newport washout from the colonic fermentation model in the presence of MccJ25, reuterin or rifampicin during 24 h was quantified by PMA-qPCR (Figure 3A). Starting at 7 log10 CFU/mL, the count dropped by a factor of 10 within 24 h in the absence of any antimicrobial agent, which was close to the theoretical washout curve over the same period of time. When MccJ25 was added to the medium, an additional nearly 10-fold drop was observed. Neither reuterin nor rifampicin had any such effect.

**FIGURE 4:**
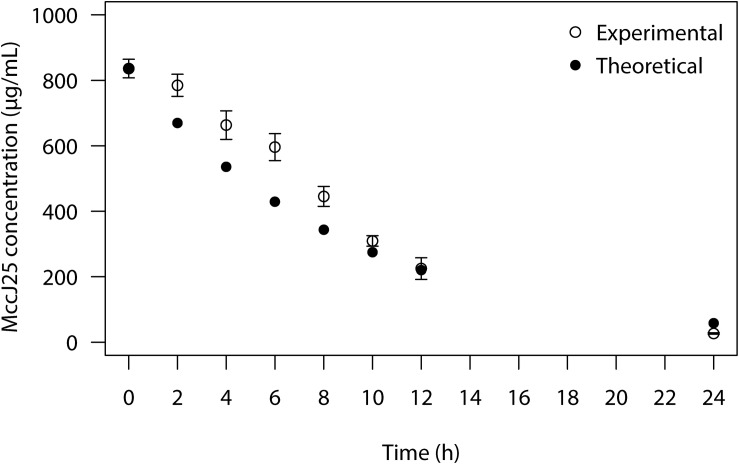
Concentration of MccJ25 over 24 h colonic fermentation in the PolyFermS system based on LC-MS quantification. (°) Experimental values measured during colonic fermentation based on the extracted ion peak areas obtained from the [M + 3H]^3+^ ion of MccJ25 (*m/z* 703.0); (🌑) Theoretical values calculated based on the dynamic flow in the PolyfermS system; measured values are means of two independent repetition experiments.

The MccJ25 bioavailability was tested in the course of the PolyFermS colon fermentation process in modified Macfarlane broth by using agar diffusion assays to measure inhibition of *Salmonella* Newport. MccJ25 produced inhibition zones varying from 1.9 to 1.2 cm in diameter (Figure 3B), which revealed it is stable and active under conditions mimicking the porcine colon. By contrast, inhibition of *Salmonella* Newport by rifampicin was weak, with an average inhibition zone diameter of 1 cm compared to the 2 cm diameter of the positive control. This activity dropped steadily during the first 12 h of fermentation and was null at 24 h. For its part, reuterin failed to produce any zone of inhibition at any time of the fermentation. Similar results were also observed when inhibitory activity of MccJ25, reuterin and rifampicin against *Salmonella* Newport were tested in the second test bioreactor of the PolyFermS system (Supplementary Figure S3). LC-MS analysis permitted to quantify MccJ25 in the complex supernatant from the PolyFermS system, confirming its stability and hence its bioavailability over the 24 h culture (Figure 4 and Supplementary Figure S4).

## Discussion

To achieve our final goal that is paving the way for further studies evaluating the potential of using MccJ25 as an alternative to antibiotics in livestock, the main objective of the present study was to assess the stability and inhibitory activity of MccJ25 under the physiological conditions encountered in the swine colon. Although *in vivo* studies conducted in mice or in model livestock animals (Forkus et al., 2017; Yu et al., 2017; Wang et al., 2020) have shown the potential of this bacteriocin as an alternative to antibiotics, and its high stability in the upper part of the GI tract has been assessed (Naimi et al., 2018), it was necessary to specifically determine the efficiency of MccJ25 against a major pathogen, such as *Salmonella* Newport, in conditions mimicking those found in the swine colon. We showed here that the potent MccJ25 inhibitory activity against *Salmonella* Newport measured in LB broth is maintained in the conditions used to mimic the physiological conditions encountered in the swine colon.

Indeed, in LB broth the MIC and MBC values determined here for MccJ25 against *Salmonella* Newport at 0.03 and 3.7 μM, respectively were in the range previously published (MIC at 0.05 μM) in an early study using a clinical isolate of *Salmonella* Newport (Rintoul et al., 2001). The MBC was 123 times higher than the MIC measured in the same conditions, which indicates that the antimicrobial activity of MccJ25 is bacteriostatic rather than bactericidal, in agreement with a previous study which reports a long-lasting bacteriostatic effect (Vincent et al., 2004). *Salmonella* Newport was significantly more sensitive to MccJ25 than to reuterin or rifampicin, which exhibited both much higher MIC and MBC values.

Numerous studies suggest that bacteriocins are effective in the treatment of infections in animal models, whether delivered as pure peptides or via bacteriocin-producing probiotic bacteria (van Winsen et al., 2001; Ojha and Kostrzynska, 2007; Riboulet-Bisson et al., 2012). In a study using a porcine model, a mixture of bacteriocin-producing probiotic strains considerably reduced infection caused by *Salmonella enterica* Serovar Typhimurium through domination in the ileum, the primary attachment site of the pathogen (Casey et al., 2007). However, the causative link between bacteriocin production and anti-*Salmonella* activity has never been clearly established. It has been further demonstrated that feeding swines with *Lactobacillus salivarius* UCC118 producing the broad-spectrum class IIb bacteriocin Abp118 affected intestinal microbiota diversity in addition to inhibiting *Salmonella*. This was attributed to competitive exclusion or immunomodulation of host defense mechanisms (Riboulet-Bisson et al., 2012). In an *in vitro* study, *Bifidobacterium thermophilum* RBL67 has been shown to inhibit colonization by *Salmonella* Typhimurium N-15 under simulated porcine intestinal conditions through a synergistic effect with selected prebiotics (Tanner et al., 2014b). Inhibition of *Salmonella* Typhimurium in swine by oral administration of microcin 24-producing *E. coli* has also been demonstrated (Frana et al., 2004). Apart from these few studies, there is no serious investigation of the behavior and stability of bacteriocins or microcins in the GI tract. Furthermore, no study has clearly established a direct link between the presence of bacteriocins and the antimicrobial activities observed in the digestive tract. Such studies require the prior development of sensitive and specific methods for the detection and quantification of bacteriocins in a complex environment such as the colon. In general, bacteriocins from Gram-negative bacteria have been less studied than those from Gram-positive bacteria and their inhibition capacities of *Salmonella* or other pathogens remain sparsely documented.

Swine colonic conditions have been reproduced previously *in vitro* using the PolyFermS model (Tanner et al., 2014a). This model allows reproducible testing of different treatments in parallel reactors, thus providing a built-in control inoculated with the same microbiota. It was used in the present study to mimic a *Salmonella* infection in the swine proximal colon and to evaluate the inhibitory activity of MccJ25 against this pathogen in such conditions (Tanner et al., 2014b). Using this model, we provide evidence of the inhibitory activity of MccJ25 against *Salmonella* Newport in the presence of the swine proximal colon conditions, including the microbiota metabolic activity. The MccJ25 antibacterial activity remained potent over 24 h of continuous fermentation, as shown by PMA-qPCR and agar diffusion assays, and was superior to those of the broad-spectrum antibiotic rifampicin and the antimicrobial compound reuterin. The inhibitory activity was shown to be correlated with the greater stability and bioavailability of MccJ25 compared to reuterin and rifampicin. Samples taken from test reactor TR1 at regular intervals over 24 h further showed significant inhibition of *Salmonella* Newport using the agar diffusion assay. The stability of MccJ25 has been demonstrated in several environments and attributed to its unique lasso structure (Rosengren et al., 2003). Known as a [1] rotaxane topology, the lasso structure confers remarkable stability at high temperatures and extreme pHs and resistance to several proteases (Salomón and Farías, 1992; Blond et al., 1999; Rosengren et al., 2003). Such features allow MccJ25 to resist very stringent conditions like those encountered in the GI tract (Naimi et al., 2018), unlike other bacteriocins such as pediocin PA-1, a class IIa bacteriocin which has been found to be sensitive to simulated GI conditions and to lose completely its antibacterial activity in the small intestine (Kheadr et al., 2010).

Reuterin represents an interesting model of antimicrobial molecule for the present study, since it has been shown to exert a broad spectrum antibacterial activity with a good activity against *Salmonella* spp. (Kuleaşan and Çakmakçı, 2002) without perturbing heavily the gut microbiota (Shornikova et al., 1997; Casas and Dobrogosz, 2000). Its failure to inhibit with efficacy *Salmonella* Newport in this study is presumably due to non-specific interactions with various compounds in the modified Macfarlane medium or possibly to instability under these conditions. Meanwhile, rifampicin was slightly inhibitory for nearly 8 h of culture, based on the agar diffusion assay but this finding was not corroborated by *Salmonella* Newport counts based on PMA-qPCR.

In this study, we succeeded for the first time in developing a specific and sensitive method using LC-MS to detect and quantify MccJ25 in a fermentation medium simulating the conditions in the porcine colon. This method allowed to confirm the bioavailability of MccJ25 and therefore makes a clear link with its anti-*Salmonella* Newport activity under such conditions. Any molecule added into the PolyFermS fermentation model is facing the colon microbiota and its metabolic activity and globally the colon conditions, which are established in the model. We have shown here that adding MccJ25 into the PolyFermS system results in significant inhibition of *Salmonella* Newport, opening up favorable prospects for the use of this bacteriocin as a novel alternative to current antibiotics. This approach using a purified bacteriocin offers several advantages compared to the use of a bacteriocin producing strain, i.e., a probiotic strain. Indeed, the implication of bacteriocins in inhibition of enteric infections by the producing bacteria remains not clearly demonstrated, as many studies report contradictory conclusions, due to a lack of sensitive and specific methods of detection and quantification of the bacteriocins produced in the GI tract and/or to the high diversity of bacteriocin producing strains and their bacteriocins (Hegarty et al., 2016). To exert the expected function, bacteriocin-producing strains must survive to the stressful conditions of the stomach and the small intestine and must further survive and multiply in the colon and produce their bacteriocins in sufficient amounts to exert their antagonistic activity. The use of purified bacteriocins allows avoiding these limitations, especially when the structure of the molecule confers it a high stability over the GI transit, as it is the case of MccJ25 whose lasso structure gives it a high resistance to both gastric pH and most proteases (Naimi et al., 2018). In the case where the bacteriocin is less stable at the GI level, the use of galenic forms allowing the protection and controlled release of the molecule would be applied.

## Conclusion

The overuse of antibiotics in livestock has raised a lot of questions in recent years. This increasingly controversial practice is pointed out in the emergence of the phenomenon of resistance and multi-resistance to antibiotics. The use of antibiotics as growth promoters has been prohibited in several countries, which makes the search for alternatives increasingly urgent. Among the alternative approaches proposed, the use of natural antimicrobials such as bacteriocins has attracted high interest. In this paper, we have shown that MccJ25 produced by *E. coli* is a potent inhibitor of *Salmonella* Newport when tested under growth conditions mimicking the swine colon environment using the PolyFermS continuous fermentation model. This bacteriocin might be considered a good alternative to antibiotics for animal feed applications, although further *ex vivo* and *in vivo* studies should be focused on validating the effectiveness of MccJ25 as an inhibitor of *Salmonella* in farmed pigs as well as evaluating its impact on the intestinal microbiota.

## Data Availability Statement

The raw data supporting the conclusions of this article will be made available by the authors, without undue reservation, to any qualified researcher.

## Ethics Statement

The collection of the swine fecal sample does not require any approval from the committee of animal research since it does not involve any animal manipulation. This study was submitted and approved by the biological risk management committee of Laval University before the start of the work. All experiments were performed in a level II laboratory. https://www.ulaval.ca/la-recherche/services-a-la-recherche-alacreation-et-a-linnovation/ethique/comite-universitaire-degestion-des-risques-biologiques.html.

## Author Contributions

SN designed and performed all the experiments, carried out data analysis and wrote the manuscript. IF managed the overall project, participated in the design of the experiments, analysis and interpretation of data, and writing of the manuscript. SR contributed to manage the overall project and to the interpretation of data and writing of the manuscript. SZ participated in the design of the experiments, performed LC-MS analysis and helped for analysis and interpretation of data and writing of the manuscript. MT participated in the preparation of the Macfarlane nutritive medium during the fermentation period. BF contributed to production and purification of reuterin and provided the swine fecal sample. MT, JT, and BF participated in the immobilization part of the fermentation procedure and assisted in the operation of the PolyFermS model.

## Conflict of Interest

The authors declare that the research was conducted in the absence of any commercial or financial relationships that could be construed as a potential conflict of interest.
