# Supplementary material for: Microcin J25 Exhibits Inhibitory Activity Against Salmonella Newport in Continuous Fermentation Model Mimicking Swine Colonic Conditions
Source: Front Microbiol. 2020 May 25;11:988. doi: 10.3389/fmicb.2020.00988 (PMC7262971; doi:10.3389/fmicb.2020.00988)
Supplement: Supplementary file 1 [file Data_Sheet_1.docx]

Supplementary Material

The antimicrobial lasso peptide microcin J25 exhibits inhibitory activity against *Salmonella* in continuous fermentation model mimicking swine colonic conditions

Sabrine Naimi, Séverine Zirah, Menel Ben Taher, Jérémie Theolier, Benoît Fernandez, Sylvie Rebuffat and Ismail Fliss*

*** Correspondence:** Corresponding Author: [ismail.fliss@fsaa.ulaval.ca](mailto:ismail.fliss@fsaa.ulaval.ca)

# Supplementary Figures and Tables

## Supplementary Tables

**Table S1.** Composition of the modified Macfarlane medium (after Tanner et al., 2014).

| **Ingredients** | **g. L^-1^ distilled water** |
| --- | --- |
| *Carbohydrates sources* |  |
| Corn starch | 4.32 |
| Pectin (citrus) | 2.00 |
| Xylan (beechwood) | 2.00 |
| Arabinogalactan (larch wood) | 2.00 |
| Guar gum | 1.00 |
| *Nitrogen-sources* |  |
| Soy peptone | 13.00 |
| Yeast extract | 4.50 |
| Mucin (from porcine stomach) | 4.00 |
| *Salts/Minerals sources* |  |
| L-cysteine HCl monohydrate | 0.80 |
| Bile extract porcine | 0.40 |
| KH_2_PO_4_ | 0.50 |
| NaHCO_3_ | 1.50 |
| NaCl | 4.50 |
| KCl | 4.50 |
| MgSO_4_ anhy. (120.37 g mol-1) | 0.64 |
| CaCl_2_ 2 H_2_O (147.02 g mol-1) | 0.15 |
| MnCl_2_ 4 H_2_O (197.91 g mol-1) | 0.20 |
| Hemin solution (0.05 g mL-1) | 0.05 |
| Tween 80 | 1.00 |

**Table S2.** Purification of MccJ25 produced by *E. coli* MC4100 pTUC202.

|  | Volume  (mL) | Protein concentration*  (mg/mL) | Total protein (mg) | Antibacterial activity  (AU/mL) | Specific activity  (AU/mg) | Recovery†  (%) |
| --- | --- | --- | --- | --- | --- | --- |
| Culture supernatant | 1500 | 0.1598 | 239.69 | 16384 | 102533 | 100 |
| Active fraction  (30% acetonitrile) | 117.8 | 0.4188 | 49.33 | 131072 | 313007 | 62.8 |
| Purified MccJ25 | 13.6 | 3.3203 | 45.16 | 1048576 | 315811 | 58.0 |

*Lowry assay †Remaining protein concentration as % of the initial concentration

## Supplementary Figures

**
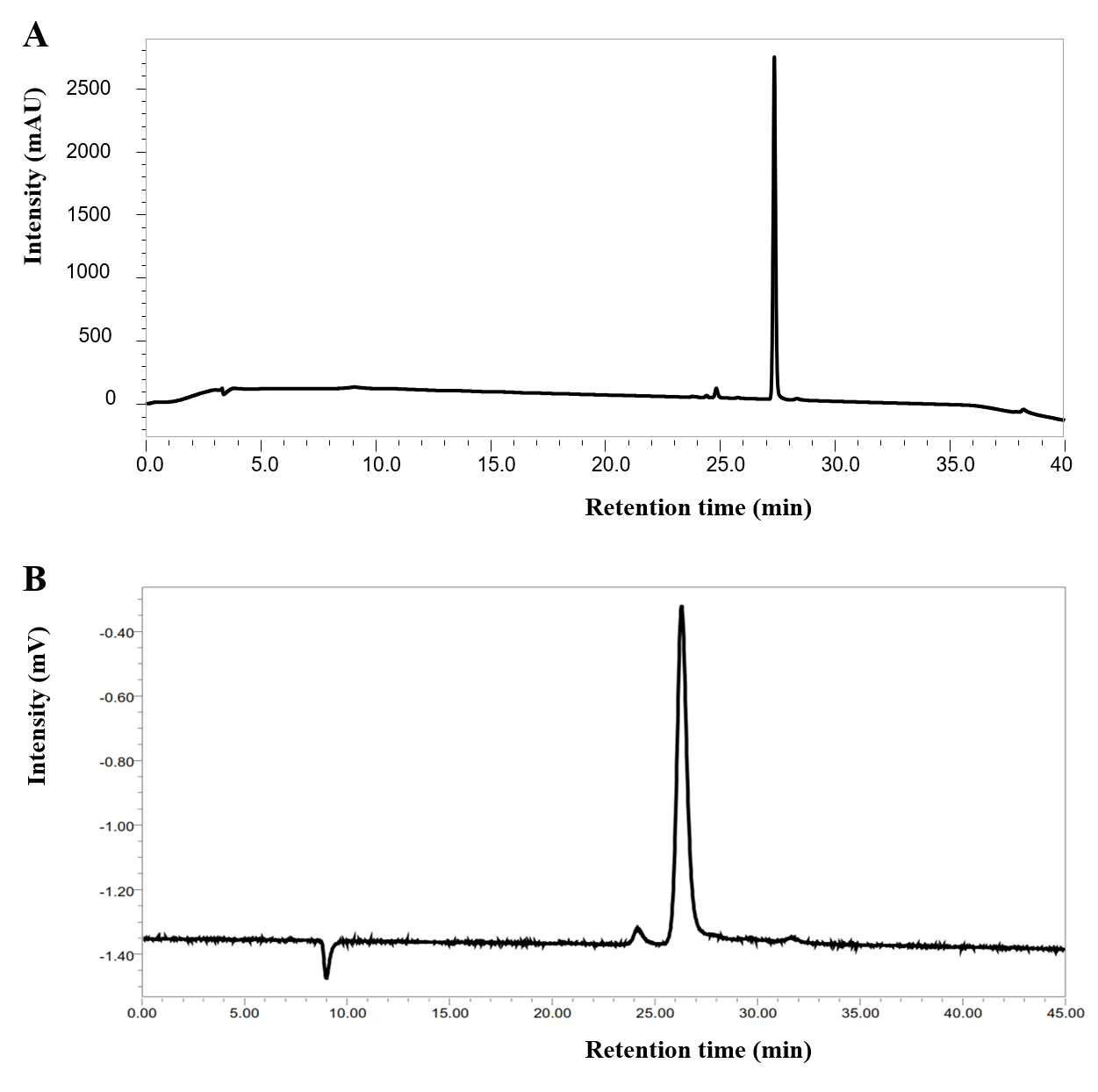
**

**Figure S1.** HPLC chromatograms of MccJ25 and reuterin. (A) RP-HPLC chromatogram of purified MccJ25 (Rt = 27.28 min) obtained using a preparative C18 column (2.1 × 25 cm) eluted with a 25–100% linear gradient of acetonitrile in 0.18% HCl in ultra-pure water at a flow rate of 10 mL/min (detection at 214 nm). (B) HPLC chromatogram of reuterin after purification (Rt = 26.2 min) obtained using a silica gel chromatography column (2.8 × 35 cm) eluted with acetone: ethyl acetate (2:1) as eluent.


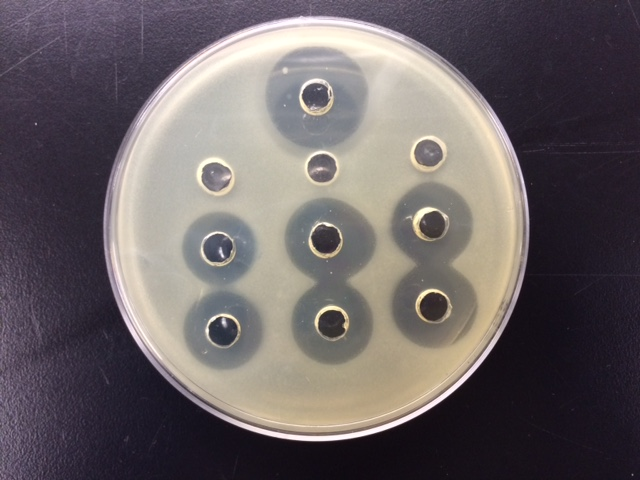

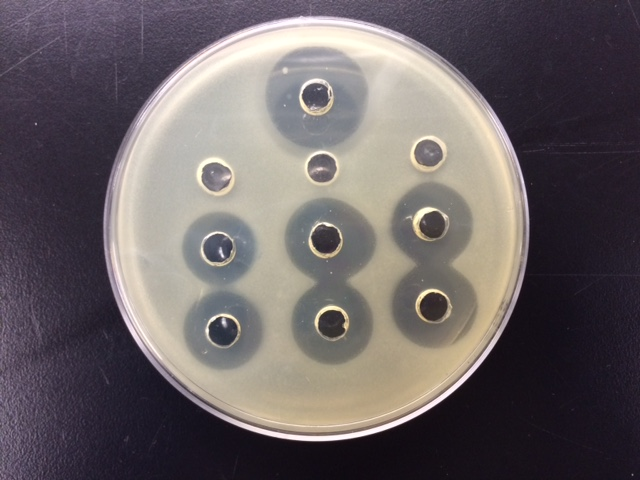

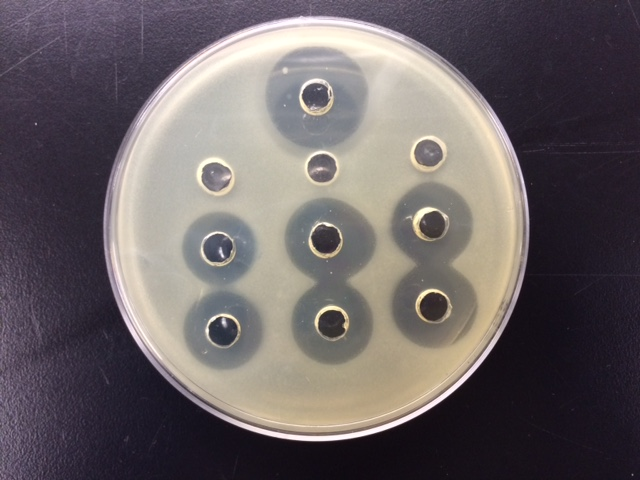

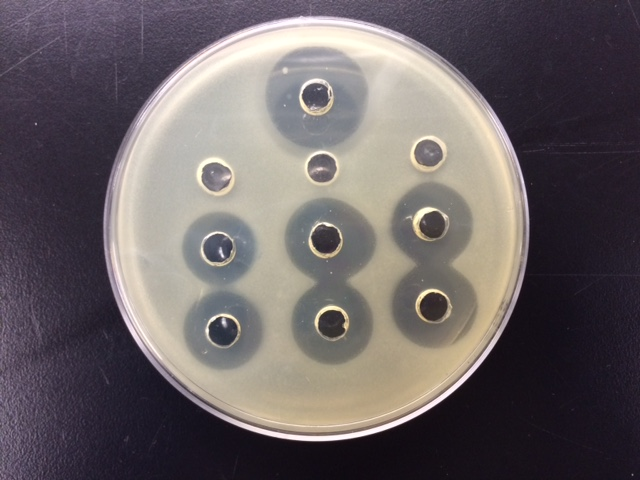

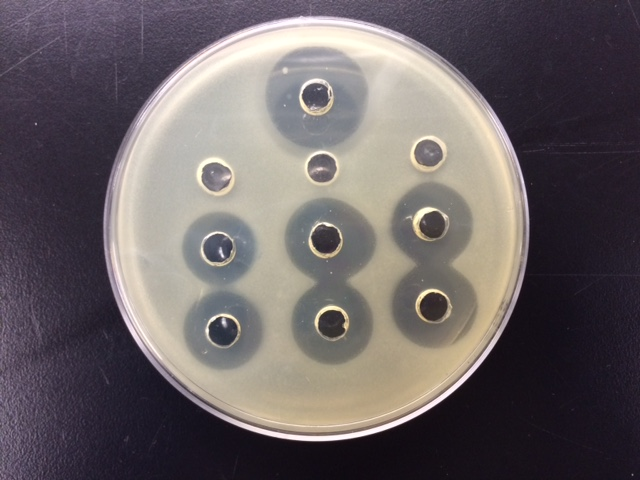

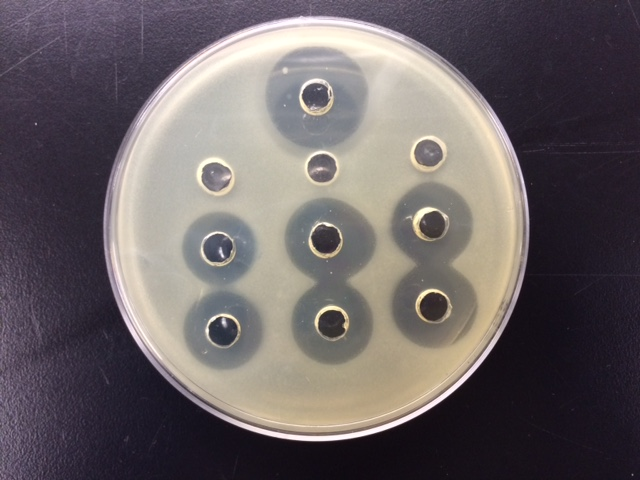

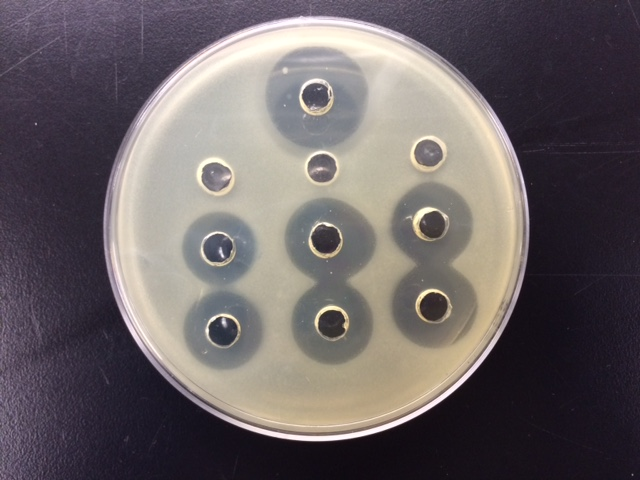


**a**

**b**

**e**

**c**

**f**

**d**

**g**

**Figure S2.** Bioavailability of MccJ25 tested at a concentration corresponding to the MBC as shown by the inhibition activity against *Salmonella* Newport in LB and modified Macfarlane media. (a) Positive control: MccJ25 diluted in ultra-pure water at a concentration of 1 mg/ml (474.6 µM), (b) and (e): negative controls: LB broth and modified Macfarlane medium without MccJ25, respectively. (c) activity of MccJ25 at a concentration of 3.71 µM corresponding to the MBC in LB broth before a 18 h incubation period, (d) activity of MccJ25 (MBC) in LB broth after 18 h incubation; (f) activity of MccJ25 (MBC) in modified Macfarlane medium before 18 h incubation, (g) activity of MccJ25 in modified Macfarlane medium after 18 h incubation.


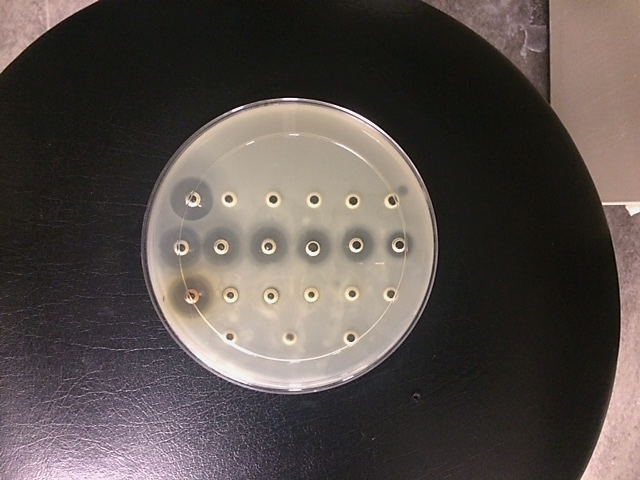


**Reuterin**

**MccJ25**

**Rifampicin**

**T^+^**

**t_0_**

**t_4_**

**t_8_**

**t_12_**

**t_24_**

**Reuterin T^-^**

**MccJ25 T^-^**

**Rifampicin T^-^**

**2 cm**

**0 cm**

**0 cm**

**0 cm**

**0 cm**

**0 cm**

**2 cm**

**1.8 cm**

**1.8 cm**

**1.6 cm**

**1.6 cm**

**1.3 cm**

**2.1 cm**

**0.8 cm**

**0.8 cm**

**0.7 cm**

**0 cm**

**0 cm**

**0 cm**

**0 cm**

**0 cm**

**Figure S3.** Agar diffusion assay showing the inhibitory activity of MccJ25, reuterin and rifampicin against *Salmonella* Newport over 24 h of culture in modified Macfarlane broth in test reactor 2 (TR2) of the PolyFermS system. T+ is the positive control (0.475 mM MccJ25)


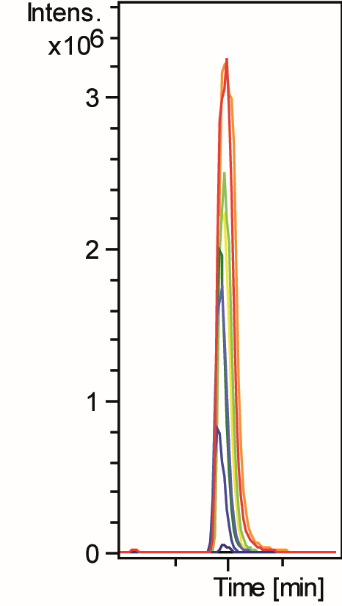


**a**

**b**


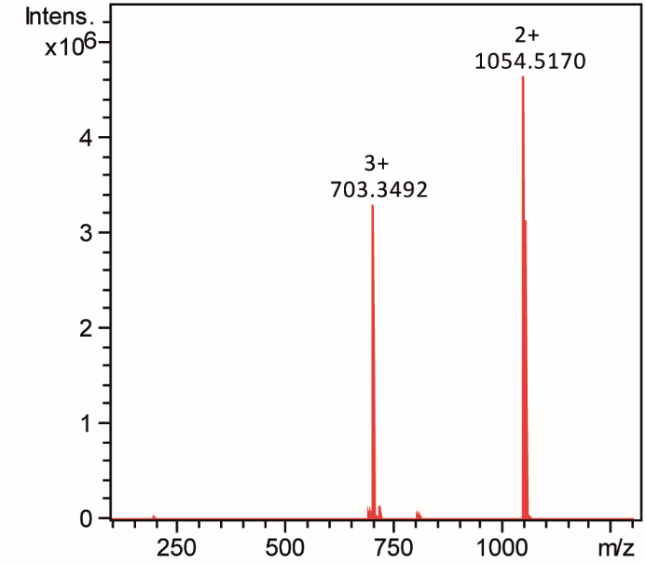


**Figure S4.** LC-MS detection of MccJ25 during the colonic fermentation in the Polyferm system. (a) Extracted ion chromatogram of the [M+3H]^3+^ ion of MccJ25 (*m/z* 703.0); (b) Mass spectrum of MccJ25 with a zoom showing the isotopic distribution of the [M+3H]^3+^ species (*m/z* 703.0).
